# Supplementary material for: Self-Assembling E2-Based Nanoparticles Improve Vaccine Thermostability and Protective Immunity against CSFV
Source: Int J Mol Sci. 2024 Jan 2;25(1):596. doi: 10.3390/ijms25010596 (PMC10778992; doi:10.3390/ijms25010596)
Supplement: Supplementary file 1 [file ijms-25-00596-s001.zip › ijms-2760438-supplementary.pdf]

Self-assembling E2-based nanoparticles improve vaccine thermostability and protective immunity against CSFV

Hetao Song[1], Sahibzada Waheed Abdullah[2], Chenchen Pei[2], Xiaoni Shi[2], Xiangyang Chen[2], Yuqing Ma[2], Shuanghui Yin[2], Shiqi Sun[2], Yong Huang[1*], Huichen Guo[2,3*]

[1]College of Veterinary Medicine, Northwest A&F University, Yangling, China

[2]State Key Laboratory for Animal Disease Control and Prevention, College of Veterinary Medicine, Lanzhou University, Lanzhou Veterinary Research Institute, Chinese Academy of Agricultural Sciences, Lanzhou, China

[3]Gansu Province Research Center for Basic Disciplines of Pathogen Biology, Lanzhou, China

* Correspondence to: Yong Huang, email: yonghuang@nwsuaf.edu.cn

Huichen Guo, email: guohuichen@caas.cn

Supplementary Figures

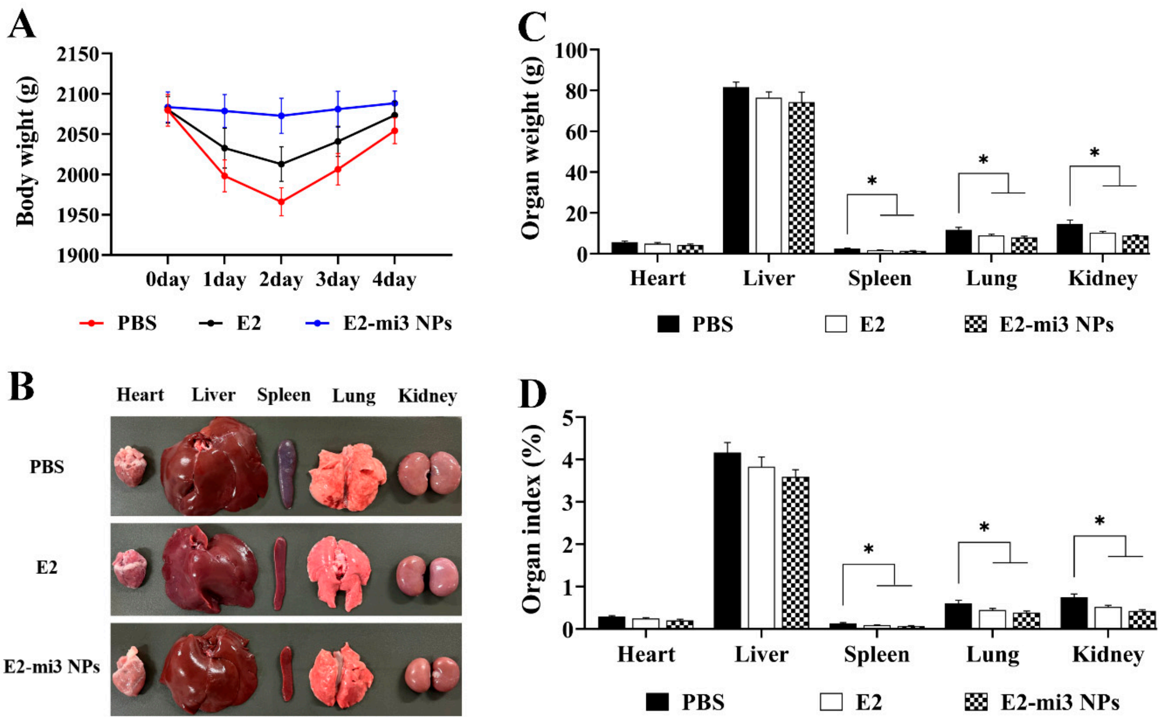

Figure S1. The changes of tissues macroscopic parameters in rabbits after C-strain challenged. (**A**) Changes in body weight of rabbits after challenged. (**B**) Representative tissues natomy changes following C-strain challenged. Organ weight (**C**) and organ index (**D**) of rabbits following challenged. Note: *$p < 0.05$.

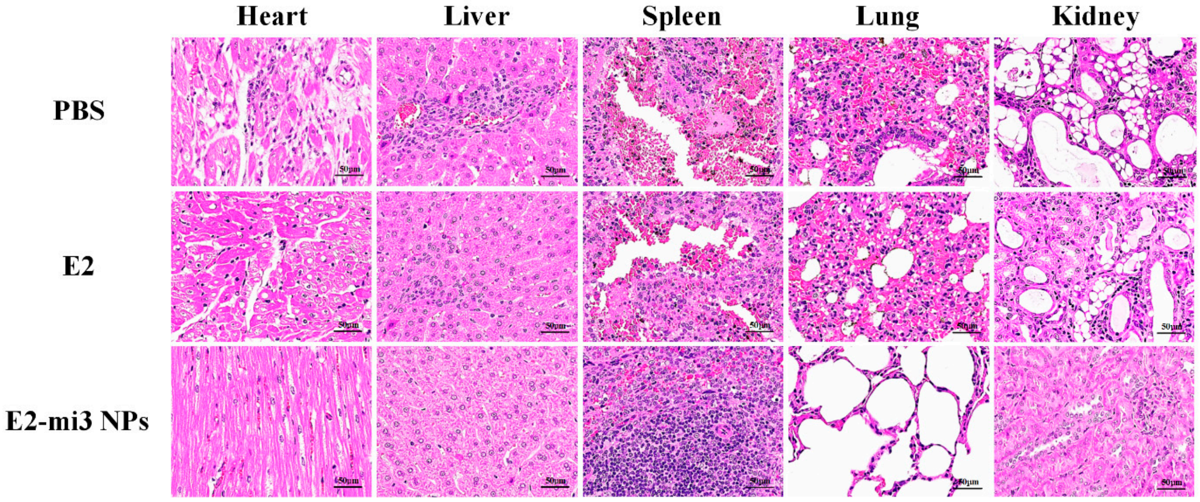

Figure S2. Representative histopathological examination of immunized rabbits challenged with CSFV C-strain by hematoxylin-eosin staining. Note: scale bar=50 μm.

Supplementary Table

Table S1 Histologic scoring

Groups	Heart	Liver	Spleen	Lung	Kidney
PBS	++	++	+++	+++	+++
E2	+	+	++	++	++
E2-mi3-NPs	—	—	—	—	—

Note: The level of severity was judged from - to ++++, which represented none to severe.
